# On the occurrence of three non-native cichlid species including the first record of a feral population of *Pelmatolapia* (*Tilapia*) *mariae* (Boulenger, 1899) in Europe

**DOI:** 10.1098/rsos.170160

**Published:** 2017-06-21

**Authors:** Juliane A. Y. Lukas, Jonas Jourdan, Gregor Kalinkat, Sebastian Emde, Friedrich Wilhelm Miesen, Hannah Jüngling, Berardino Cocchiararo, David Bierbach

**Affiliations:** 1 Department of Biology and Ecology of Fishes, Leibniz Institute of Freshwater Ecology and Inland Fisheries, Mueggelseedamm 310, 12587 Berlin, Germany; 2 Faculty of Life Sciences, Humboldt University of Berlin, Invalidenstrasse 42, 10115 Berlin, Germany; 3 Department of River Ecology and Conservation, Senckenberg Research Institute and Natural History Museum Frankfurt, Clamecystrasse 12, 63571 Gelnhausen, Germany; 4 Institute for Ecology, Evolution and Diversity, Goethe-University, Max-von-Laue-Str. 13, 60438 Frankfurt/M, Germany; 5 Senckenberg Biodiversity and Climate Research Centre, Senckenberg Gesellschaft für Naturforschung, Senckenberganlage 25, 60325 Frankfurt/M, Germany; 6 Zoologisches Forschungsmuseum Alexander Koenig, Sektion Ichthyologie, Adenauerallee 160, 53113 Bonn, Germany; 7 Senckenberg Research Institute and Natural History Museum Frankfurt, Conservation Genetics Group, Clamecystrasse 12, 63571 Gelnhausen, Germany

**Keywords:** biological invasion, non-native species, thermally influenced freshwater systems, thermally polluted, invasion biology, tilapia

## Abstract

Thermally influenced freshwater systems provide suitable conditions for non-native species of tropical and subtropical origin to survive and form proliferating populations beyond their native ranges. In Germany, non-native convict cichlids (*Amatitlania nigrofasciata*) and tilapia (*Oreochromis* sp.) have established populations in the Gillbach, a small stream that receives warm water discharge from a local power plant. Here, we report on the discovery of spotted tilapia (*Pelmatolapia mariae*) in the Gillbach, the first record of a reproducing population of this species in Europe. It has been hypothesized that *Oreochromis* sp. in the Gillbach are descendants of aquaculture escapees and our mtDNA analysis found both *O. mossambicus* and *O. niloticus* maternal lineages, which are commonly used for hybrids in aquaculture. Convict cichlids and spotted tilapia were most probably introduced into the Gillbach by aquarium hobbyists. Despite their high invasiveness worldwide, we argue that all three cichlid species are unlikely to spread and persist permanently beyond the thermally influenced range of the Gillbach river system. However, convict cichlids from the Gillbach are known to host both native and non-native fish parasites and thus, non-native cichlids may constitute threats to the native fish fauna. We therefore strongly recommend continuous monitoring of the Gillbach and similar systems.

## 1. Introduction

In colder regions of the world, many freshwater systems are thermally influenced, i.e. show water temperatures above the normal range [1]. In addition to sites that receive warm water by geothermal sources (e.g. [2–8]), thermal conditions are often altered by anthropogenic activities such as discharges of heated waters from power plants or the dewatering of mines (e.g. [9–18]). These thermally influenced freshwater (TIF) systems are hotspots for non-native organisms, as they provide suitable habitats for species of tropical and subtropical origin (e.g. [2–4,7–15,17,19–25]). While biological invasions are known to be a major driver of species extinctions and biodiversity loss [26–30], the importance and influence of TIFs remain mostly understudied.

The release of unwanted pets by aquarium hobbyists has been assumed as the main introduction pathway for non-native species into TIFs [17,31–37]. For example, aggressive behaviour, rapid reproduction, large size and illness are factors that increase the likelihood of aquarium fish to be released into the wild [31,38,39]. However, also introductions by aquaculture escapees are documented (e.g. [15,40–42]). The resulting artificial communities in TIFs [43,44] often comprise native as well as non-native species of both invertebrates (e.g. crustaceans; [13,21,45]) and vertebrates (e.g. fish; [4,8,15]).

One of the TIFs in central Europe is the Gillbach near the city of Cologne in Germany. This stream receives warm water effluents year round from a lignite power plant. Near the influx water temperatures rarely drop below 19°C, whereas 2 km downstream a minimum of 13°C was reported (February 2012; [15]). These conditions allowed several non-native tropical and subtropical fish species like *Ancistrus* sp.*, Poecilia reticulata* and *Pseudorasbora parva* as well as some invertebrates (*Neocaridina davidi* and *Macrobrachium dayanum*) and tropical plants (*Vallisneria spiralis*) to establish self-sustaining populations [13,15,46]. Most of these are popular ornamental species, making an introduction via aquarium release the most probable invasion pathway and plausible scenario for the Gillbach.

Our current paper focuses on members of another (sub)tropical fish family inhabiting the Gillbach: Cichlidae. All members of this taxonomic family stem from the tropics or subtropics and many of them have been dispersed worldwide over the past century as a result of intentional introductions. Larger predatory cichlids (e.g. *Cichla ocellaris, Cichlasoma managuense, Serranochromis robustus;* [47]) are selected for stock enhancement, whereas certain omnivorous and herbivorous species are used as agents in the control of aquatic weeds (e.g. *O. aureus* [48], *Coptodon zillii* [49]), disease vector insects (e.g. *O. mossambicus* [50]) or nuisance molluscs (*Astatoreochromis alluaudi* [51], *Coptodon rendalli* [52]).

The most famous representatives of this family are commonly known and collectively referred to as ‘tilapia’ (genera *Sarotherodon, Oreochromis* and *Tilapia*; *sensu* Trewavas [53]) (e.g. [54–56]). According to Canonico *et al*. [55] most introductions of these genera have occurred due to aquaculture activities. In fact, the farming of tilapia (*Oreochromis* spp.) is currently the most widespread type of aquaculture in the world and only second to carp by volume of production [57]. In 1998, first specimens of a tilapia hybrid have been reported for the Gillbach and were identified as *O. niloticus *×* mossambicus* based on live coloration [41]. As most of today's tilapia culture is based on hybrids (most often between *O. niloticus, O. aureus* and *O. mossambicus;* [58]), we used DNA analysis, alongside classical morphological analysis, for species identification in our current study.

Besides their use in aquaculture, cichlids are also very popular with aquarists as they show a rich array of coloration and behavioural displays. Some species (e.g. *Aequidens pulcher, Amatitlania nigrofasciata, Astronotus ocellatus, Cichlasoma* spp., *Geophagus brasiliensis, Hemichromis letourneauxi, Sarotherodon melanotheron*; [59]) have been transported widely around the world via the aquarium trade and many introductions have been the result of occasional releases from home aquaria or stock disposal from dedicated breeding facilities of the aquarium trade. One of the most popular species within the ornamental trade is the convict cichlid (*Amatitlania nigrofasciata*), originally stemming from Central America. So far, the only stable population of *A. nigrofasciata* in Germany seems to be established in the Gillbach and was first described in 1998 [17,41].

**Figure 1.**
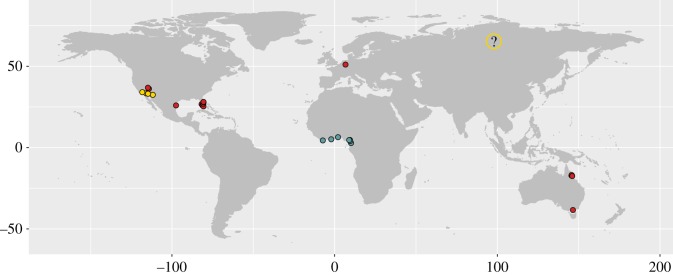
Distribution of *Pelmatolapia mariae. P. mariae* has been introduced beyond its natural range in West Africa (blue), with established populations in Australia, USA and Germany (red). Note that some records of *P. mariae* are location unspecific (indicated by question mark) or are suspected of having been subject to misidentification (yellow). For more detailed information on specific introduction sites of *P. mariae* refer to Bradford *et al*. [60] and Nico & Neilson [61].

In the current paper, we first report on the occurrence of another reproducing cichlid species in the Gillbach, which we identified as the spotted tilapia, *Pelmatolapia mariae.* With the new record of *P. mariae* (figure 1), the Gillbach now seems to harbour stable populations of at least two large African cichlids (*Oreochromis* sp. and *P. mariae*) and one Central American cichlid (*A. nigrofasciata*)—all of which have a long invasive history all over the world [55].

## 2. Material and methods

### 2.1. Study system

**Figure 2.**
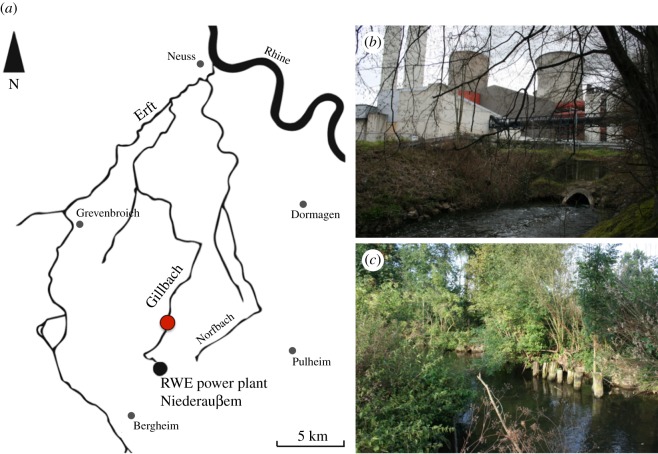
(*a*) Map of the Gillbach and its position within the Rhine catchment. Both the locations of the temperature logger placed by the inlet of the RWE power plant Niederaußem (black; *b*) and the sampling site near Rheidt (red; *c*) are indicated.

The Gillbach is a 28 km long stream within the Erft drainage, part of the Rhine basin of central Europe (figure 2*a*). The river flows through the North Rhine Lignite field in Germany, a hub for opencast mining and electrical energy industries. Its original headwaters being destroyed, it is now fed solely by the warm water discharge of the coal-fired power plant ‘Niederaußem’ (50°59'46.82′′ N, 6°39'50.56′′ E, RWE Power Inc.; figure 2*b*) located west of Cologne. At the site near Hüchelhoven/Rheidt (51°00'39.5′′ N, 6°41'02.1′′ E), the stream has been straightened to accommodate agriculture and developmental needs. The streambed of the Gillbach (approx. 3 m wide and 30–80 cm deep) consists almost entirely of artificially placed rocks as well as sand and mud. Owing to the coverage by bushes and trees, submerged vegetation is mostly absent (figure 2*c*).

### 2.2. Sampling

The water temperature at the stream's inlet was recorded every 4 h using an Onset HOBO data logger for the period of 19 March 2016 until 4 May 2017. Two kilometres downstream of this site, voucher specimens were caught using seine-netting (mesh size 6 mm) in September 2016. All specimens were immediately euthanized with an overdose of clove oil and preserved in 99% ethanol for further genetic analysis. Morphological species determination of all African cichlids was performed using standard keys (table 1; [53,62–64]). The identification of the Central American cichlid *A. nigrofasciata* followed Schmitter-Soto [65,66]. All native fish species were identified according to Kottelat & Freyhof [67]. Afterwards, all specimens were integrated into the ichthyologic collection at the Zoologisches Forschungsmuseum Alexander Koenig (ZFMK) in Bonn, Germany under the project numbers Lukas_Gillbach2016_01 to Lukas_Gillbach2016_15.

### 2.3. DNA extraction and mitochondrial DNA analysis

We extracted DNA from fin-clips of 14 fish specimens using the QIAGEN Blood and Tissue Kit (QIAGEN GmbH, Germany) as recommended by manufacturer's instructions. All extracts were measured with the NanoDrop™ Spectrophotometer (Thermo Fisher Scientific, Waltham, MA, USA) and normalized to a concentration of approximately 10 ng µl^−1^ for further analysis. We analysed partial sequences of two mitochondrial DNA genes using the following primer pairs (for/rev): 1. Cytochrome b (Cyt *b*) L14734 (5′-AACCACCGTTGTTATTCAACT-3′) and H15557 (5′-GGCAAATAGGAARTATCAYTC-3′); 2. Cytochrome c oxidase subunit 1 (COI) L6199 (5′-GCCTTCCCWCGAATAAATAA-3′) and H6855 (5′-AGTCAGCTGAAKACTTTTAC -3′) [68]. PCR reactions were carried out in a total reaction volume of 15 µl, including 3 µl template, 3 mM MgCl2, 1X standard Taq (Mg-free) reaction buffer, 0.2 mM of each dNTP, 0.3 µM of each primer and 0.66 units Taq polymerase (New England BioLabs GmbH, Germany). We used the following thermal cycling parameters: 3 min initial denaturation at 95°C followed by 35 cycles (30 s at 94°C, 30 s at 54°C and 60 s at 72°C) plus a final extension step of 30 min at 72°C. PCR products were purified with a mixture of five units Exonuclease I (Thermo Scientific, Waltham, MA, USA) and 16 units of FastAP™ Thermosensitive Alkaline Phosphatase (Thermo Scientific). Sequencing was performed in both directions with PCR primers using the BigDye Terminator 3.1 sequencing kit (Life Technologies GmbH, part of Thermo Fisher Scientific) with an initial denaturation step of 60 s at 95°C, followed by 30 cycles of 10 s at 96°C, 10 s at 50°C and 120 s at 60°C. Products were purified with ABI-XTerminator beads (Life Technologies GmbH, part of Thermo Fisher Scientific) and separated on an ABI 3730 DNA Analyzer (Life Technologies GmbH, part of Thermo Fisher Scientific). The obtained sequence data were analysed in GENEIOUS 7.1.9 (Biomatters) and blasted for species determination using default settings in the National Centre for Biotechnology Information (NCBI) GenBank. The resulting sequences were submitted as Blast queries to Genbank. COI sequences were deposited in GenBank under accession numbers KY565238–KY565240 and Cyt *b* sequences can be accessed under KY582461–KY582463 (table 2).

## 3. Results

**Figure 3.**
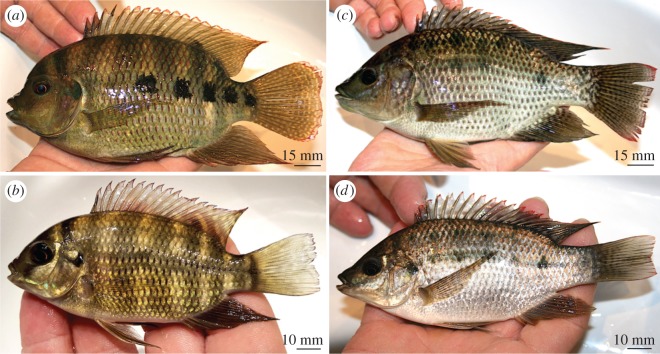
Live coloration of caught African cichlids. (*a*) *Pelmatolapia mariae*, adult male SL 154 mm; (*b*) *Pelmatolapia mariae*, juvenile SL 56 mm; (*c*) *Oreochromis* sp., adult female SL 160 mm; (*d*) *Oreochromis* sp., juvenile SL 92 mm.

**Figure 4.**
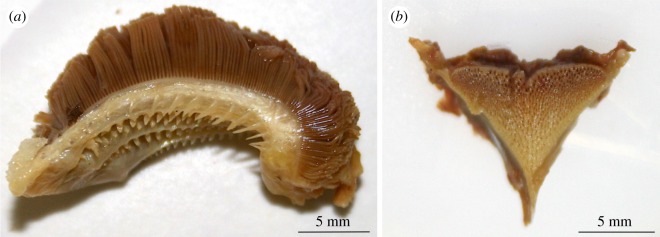
Anatomical features of *Pelmatolapia mariae*. A maximum of 15 gill rakers on lower limb of isolated first gill arch (*a*). The LPJ (*b*) is triangular with blade shorter than toothed section.

We first observed individuals of *Pelmatolapia mariae* near Rheidt in August 2016 (video observations, see the electronic supplementary material). In September 2016, we captured six specimens of *P. mariae* including two juveniles (figure 3*b*) and four adults (figure 3*a*). These ranged in size from 56 to 154 mm standard length (SL; table 1). All showed typical morphological features (table 1) and the distinctive coloration of *P. mariae* (e.g. ventral bars in juveniles (figure 3*b*) and dark caudal spots in adult specimens (figure 3*a*)). Furthermore, we counted a maximum of 15 gill rakers on the lower limb of the first branchial arch (figure 4*a*). Analysis of the lower pharyngeal jaw (LPJ; figure 4*b*) showed that its ventral keel was shorter than the toothed section. Both features are in accordance with the species' description by Teugels & Thys van den Audenaerde [62,63]. Molecular analysis of the mitochondrial COI gene confirmed the identity of *P. mariae* (100% matching with accession number KJ669646.1).

**Table 1.** Basic data on specimens of African cichlid species collected in the Gillbach stream, including numbers of individuals captured, sex, size (standard length, SL) and meristic information. Reference values follow Teugels & Thys van den Audenaerde [62] for *P. mariae* and Trewavas [53] for *Oreochromis* spp. Note that fin ray counts can differ between localities [60].

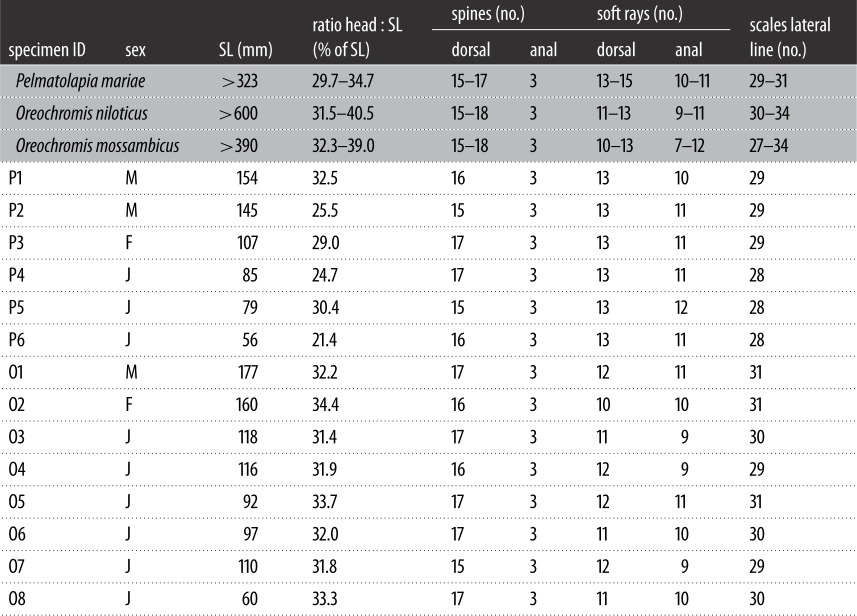

We caught eight specimens of the genus *Oreochromis.* Our morphological analysis found all individuals to show overlapping morphological characteristics of both *O. mossambicus* and *O. niloticus* (table 1) and a more *O. mossambicus*-like coloration and body shape (figure 3*c,d*; reddish fins, elongated snout, no bars at caudal fin typical for *O. niloticus*). However, our molecular analysis found both mitochondrial lineages of *O. mossambicus* (species O1, O3, O5–O8, matching with 99% identity in all cases; table 2) as well as *O. niloticus* (species O2 and O4, matching with greater than or equal to 96% in all cases; table 2), indicating that both maternal lineages were once introduced (*O. mossambicus* and *O. niloticus* and/or their hybrids). The wide range of sizes indicates that the *Oreochromis* population is breeding in the Gillbach. In fact, an adult female was mouthbrooding newly hatched fry at the point of capture.

**Table 2.** Molecular species identification. Processed samples of ‘tilapia’ species included in this study and top matches from GenBank database. Maximum identity percentage of the sequences refers to pair-wise alignments with the closest match (n.a. identifies no match greater than 90%).

	GenBank accession number		best BLAST hit with GenBank accession number and maximum identity percentage		
specimen ID	COI	Cyt b	COI	Cyt b	identified species
P1	KY565240	KY582463	KJ669646.1 (100%)	n.a.	*P. mariae*
P2	KY565240	KY582463	KJ669646.1 (100%)	n.a.	*P. mariae*
P3	KY565240	KY582463	KJ669646.1 (100%)	n.a.	*P. mariae*
P4	KY565240	KY582463	KJ669646.1 (100%)	n.a.	*P. mariae*
P5	KY565240	KY582463	KJ669646.1 (100%)	n.a.	*P. mariae*
P6	KY565240	KY582463	KJ669646.1 (100%)	n.a.	*P. mariae*
O1	KY565238	KY582461	AY597335.1 (99%)	AY597335.1 (99%)	*O. mossambicus*
O2	KY565239	KY582462	GU370126.1 (98%)	GU477628.1 (97%)	*O. niloticus*
O3	KY565238	KY582461	AY597335.1 (99%)	AY597335.1 (99%)	*O. mossambicus*
O4	KY565239	KY582462	GU370126.1 (98%)	GU477628.1 (97%)	*O. niloticus*
O5	KY565238	KY582461	AY597335.1 (99%)	AY597335.1 (99%)	*O. mossambicus*
O6	KY565238	KY582461	AY597335.1 (99%)	AY597335.1 (99%)	*O. mossambicus*
O7	KY565238	KY582461	AY597335.1 (99%)	AY597335.1 (99%)	*O. mossambicus*
O8	KY565238	KY582461	AY597335.1 (99%)	AY597335.1 (99%)	*O. mossambicus*

A third cichlid species, the convict cichlid (*Amatitlania nigrofasciata*) was also caught and we observed many breeding pairs in shallow areas along the stream. In addition, two native European chub (*Squalius cephalus*), one native barbel (*Barbus barbus*), as well as one specimen of the tropical *Ancistrus* sp*.* were caught. Assignment of armoured catfish specimens further than the genus *Ancistrus* remains tentative until systematics are further resolved [69].

**Figure 5.**
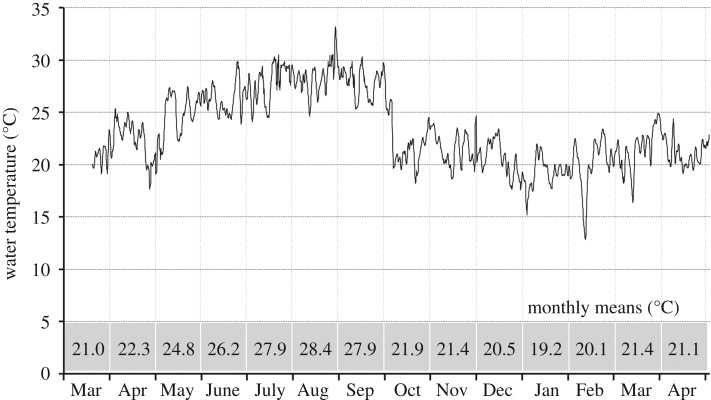
Daily water temperatures in the Gillbach in 2016/2017. Fluctuations in the daily means (six data points per day) were recorded for the source of the Gillbach from March 2016 to May 2017 and monthly means were calculated (bottom panel).

Our temperature measures largely confirmed previous measures [15] and water temperatures never dropped below 8.38°C (only three readings were below 12.5°C). Monthly means at the Gillbach (figure 5, bottom panel) ranged from 19.2°C (January 2017) to 28.4°C (August 2016). Reference measurements taken at the sampling site in Hüchelhoven revealed an average temperature difference of ±2 K compared with the stream's source.

## 4. Discussion

The Gillbach near Cologne represents a thermal refuge, which provides suitable conditions for a variety of introduced non-native species. In fact, the cichlids *O. niloticus* × *O. mossambicus, A. nigrofasciata* and *Maylandia* (*Pseudotropheus*) *aurora* have been reported for the Gillbach/Erft system during previous samplings [15,17,41]. The occurrence of *M. aurora* could not be confirmed after 1998, but our sampling now adds another species to that list: the West African spotted tilapia *P. mariae*.

Our mtDNA analysis found both *O. mossambicus* and *O. niloticus* maternal lineages. Sequencing of the Cyt *b* and COI gene, however, does not enable us to clearly detect hybrids. Nevertheless, found specimens from the Gillbach largely share the same morphological phenotype corroborating the presence of a hybrid population. *Oreochromis* species hybridize well when occurring syntopically [58] and the use of hybrids is common practice in aquaculture. The Gillbach's *Oreochromis* sp. are assumed to be (descendants of) escapees from a closed aquaculture facility that employed the power plant's warm water discharge for its production [41]. We do not know whether both species (*O. niloticus* and *O. mossambicus*) were initially introduced and hybridized afterwards in the Gillbach or whether hybrids were initially released. Samplings in 2012 [15] and 2016 (present study) found both adults and juveniles, thus, *Oreochromis* sp. can be considered to have established a reproducing population within the Gillbach.

*Amatitlania nigrofasciata* have successfully persisted in the Gillbach for more than 18 years now (first record by Höfer & Staas [41]). Individuals of different size classes were plentiful during samplings in 1998 [41], 2012 [15], 2014 [17] and 2016 (present study). Established wild populations are known for Asia, the Middle East, North and Central America and Australia [59,70–73]. So far the only other introduction sites within Europe are two thermal refugia in Italy [4] and Austria [3], both of which are very similar to the Gillbach system in their habitat characteristics and species assemblage.

To this day, *P. mariae* has been introduced to at least three continents (figure 1; [59–61]). Records from Australia show an established population in the cooling waters of a power station [74] very similar to the Gillbach system. The Gillbach, with its sandy streambed and shallow littoral zones, allows for conditions that correspond to *P. mariae*'s natural habitats in Nigeria [75]. Submerged vegetation is mostly absent in this section of the Gillbach; however, artificially placed rocks may be suitable as spawning substrate [76] and provide shelter during the larval and juvenile stages, which are most prone to predation [77]. Mature individuals have virtually no predators in the Gillbach, but eggs and larvae are most probably cannibalized or preyed on by bigger *Oreochromis* sp., as well as native species, such as European chub and Common barbel. The dental and gill morphology of the spotted tilapia allows for foraging behaviour that includes both plankton-filtering and grazing [78,79] and thus *P. mariae* finds suitable conditions in the Gillbach for a diet dominated by plant material [17]. Moreover, the species' documented tolerance to a wide range of temperatures, salinity and dissolved oxygen concentrations [60], not only fosters its dispersal, but also makes it a potential candidate for aquaculture [80,81]. However, the spotted tilapia is an available aquarium fish and its first occurrence in the Gillbach almost two decades later than the closure of the aquaculture facility renders a release by aquarists the most likely reason for the introduction of the species. Similar introduction pathways are known from certain locations in Australia [82–84] and North America ([85,86]; figure 1).

As all cichlid species currently present in the Gillbach stem from the tropics and subtropics, they cannot cope with water temperatures commonly encountered during harsh German winters. Several studies report on the temperature ranges in their natural habitats (17°C–35°C for *O. mossambicus* and 13.5°C–33°C for *O. niloticus* [87]; 20°C–36°C for *A. nigrofasciata* [88]; 20°C–25°C for *P. mariae* [89]). However, extended temperature ranges have been shown for introduced populations of *Oreochromis* spp. [87] and *P. mariae* [90,91] with lethal limits of all three species being reported to be below 11.5°C [92–94]. While average monthly temperatures in the Gillbach never dropped below 19°C (figure 5), we did record temperature spikes (less than 4 h) down to 9.5°C (26 April 2016) and even 8.4°C (30 April 2016, 2 January 2017). However, fish originating from the ornamental trade are often more cold-tolerant than their wild-type counterparts (e.g. [15] for *Poecilia reticulata*; [14] for *Xiphophorus variatus*). In fact, several studies showed certain tilapias (*Oreochromis* spp.) are capable of surviving in rapid temperature fluctuations down to 10°C with seemingly no detrimental effect [9,95,96].

The Gillbach drains into the Erft river, which is equally influenced by the effluents of nearby power plants (e.g. RWE power plant ‘Frimmersdorf’; [97,98]) and mine dewatering (Lignite mining area ‘Garzweiler’). Temperatures in 2016 never dropped below 10°C (e.g. February near Glesch; [99]) and tropical non-natives such as *Poecilia reticulata* and even piranhas have been found here regularly (Udo Rose 2007, personal communication). The Erft drains into the Rhine, which is currently the most thermally polluted river in the world [1] with a high richness and abundance of non-native species [100,101]. Nevertheless, temperatures in the Rhine sometimes drop to below 4°C (e.g. January 2017 near Düsseldorf-Flehe; [99]) and thus most non-natives with tropical or subtropical origin would not survive outside the areas affected by warm water influx. In fact, both Deacon *et al*. [102] and Jourdan *et al*. [15] suggested that an expansion of the tropical guppy into adjacent, not artificially heated streams is unlikely.

One often neglected risk emanating from non-native species is their ability to distribute non-native pathogens [103,104]. Emde *et al*. [17] demonstrated that TIFs may function as reservoirs for non-native pathogens and parasites. The authors found convict cichlids from the Gillbach to serve as both intermediate and final host for one native (*A. anguillae*) and three introduced fish parasite species (*A. crassus*, *B. acheilognathi*, *C. cotti*), thereby increasing the risk of spread of these parasites beyond their current distribution. First samples of *Oreochromis* sp*.* indicate that this species plays no significant role in the spread of parasites within the Gillbach system due to its mainly plant-based diet (Sebastian Emde 2016, personal communication). Whether *P. mariae* constitutes a greater threat in this regard should be in the focus of future investigations as its diet differs from that of *Oreochromis* sp. In its native range, *P. mariae* carries heavy parasite loads with a large proportion of the population being infected (greater than 50%; [105–107]).

The Gillbach exemplifies that TIFs can accumulate more and more non-native species over time [13,15,41,46]. Observations from the Warmbach near Villach (Austria) provide similar results: each consecutive sampling found new non-native species (2001: *Hemichromis letourneauxi*; 2002: *Hemichromis fasciatus;* 2005: *Procambarus clarkia, Lepomis gibbosus, Poecilia reticulata, Xiphophorus maculatus, Xiphophorus hellerii*; 2007: *Amatitlania nigrofasciata, Oreochromis mossambicus, Ancistrus dolichopterus, Maylandia aurora, Hyphessobrycon erythrostigma* [3,108]). Overall, greater effort in prevention of the release of non-native species is required to stop the spread outside their native range through a raising of awareness in fish keepers and society alike.

## 5. Conclusion

(i) The Gillbach—a TIF that has accumulated non-native species over time—is now harbouring stable populations of three cichlid species. We confirm the occurrence of *A. nigrofasciata* and *Oreochromis* sp., both of which have been previously described for this system. In fact, molecular analyses of *Oreochromis* specimens identified the existence of mitochondrial lineages of *O. mossambicus* and *O. niloticus*. We further report on the occurrence of *P. mariae,* which is the first record of this species in Europe.
(ii) Cichlids in TIFs may play a role in disease and parasite transmission. It has been shown that convict cichlids from the Gillbach serve as hosts for both non-native and native parasites. Thus, we strongly recommend further investigations on the potential transmitter role of *Oreochromis* sp. and *P. mariae* in the Gillbach system.
(iii) We urgently call for an inclusion of TIFs into continuous monitoring programmes. The Gillbach provides a fruitful system to study invasion processes in detail and improve our understanding of potential impacts on native species and ecosystems. We further prompt that raising public awareness is much needed. While there are several scientific publications on the Gillbach's non-native fish fauna, alien species databases either show outdated records [109,110] or no records of any introduced cichlids in Germany [111,112] as of May 2017. Furthermore, we urge fish keepers to refrain from releasing their pets into ‘suitable’ habitats (which is already prohibited by the German Animal Welfare Act [113]; §3 Abs. 3, 4 TierSchG).

## Data accessibility

Our data are deposited at Dryad (http://dx.doi.org/10.5061/dryad.sd7vh; [114]). mtDNA sequences are available at the NCBI GenBank under the following accession codes: KY565238–KY565240 and KY582461–KY582463. All specimens were integrated into the ichthyologic collection at the ZFMK in Bonn, Germany under the project numbers Lukas_Gillbach2016_01 to Lukas_Gillbach2016_15.
